# Supplementary material for: ATG gene duplication in vertebrates: evolutionary divergence and its functional implications
Source: Autophagy. 2026 Jan 24;22(4):648–65. doi: 10.1080/15548627.2026.2618126 (PMC13020874; doi:10.1080/15548627.2026.2618126)
Supplement: ATG_duplication_Supplementary_Figures_and_Tables_20251208.docx [file KAUP_A_2618126_SM5070.docx]

***ATG* gene duplication in vertebrates: evolutionary divergence and its functional implications**

Sidi Zhang^1,†^, Ikuko Koyama-Honda^1^, Daiki Hiratsuka^1,2^, Noboru Mizushima^1,†^

^1^ Department of Biochemistry and Molecular Biology, Graduate School of Medicine, The University of Tokyo, Tokyo, Japan

^2^ Present address: Department of Obstetrics and Gynecology, Graduate School of Medicine, The University of Tokyo, Tokyo, Japan

^†^ Correspondence to: S. Zhang (sidizhang@m.u-tokyo.ac.jp) and N. Mizushima (nmizu@m.u-tokyo.ac.jp)

**This file includes:**

Tables S1–S3

Figures S1–S4

**Table S1**. List of 30 Chordata species and five outgroup species used in the homology search and protein sequence evolution analysis.

| **Taxonomy group** | **Species** | **Common name** | **Genome id** |
| --- | --- | --- | --- |
| Cephalochordata | *Branchiostoma lanceolatum* | European lancelet | GCA_927797965.1 |
| Cephalochordata | *Branchiostoma floridae* | Florida lancelet | GCF_000003815.2 |
| Urochordata | *Ciona intestinalis* | Sea squirt | GCF_000224145.3 |
| Cyclostomata | *Eptatretus burgeri* | Inshore hagfish | GCA_900186335.2 |
| Cyclostomata | *Eptatretus atami* | Brown hagfish | Marlétaz et al. 2024 [33] |
| Cyclostomata | *Petromyzon marinus* | Sea lamprey | GCF_010993605.1 |
| Cyclostomata | *Lethenteron reissneri* | Far Eastern brook lamprey | GCF_015708825.1 |
| Cartilaginous fish | *Callorhinchus milii* | Australian ghostshark | GCF_018977255.1 |
| Cartilaginous fish | *Carcharodon carcharias* | Great white shark | GCF_017639515.1 |
| Cartilaginous fish | *Leucoraja erinacea* | Little skate | GCF_028641065.1 |
| Ray-finned fish | *Danio rerio* | Zebrafish | GCF_000002035.6 |
| Ray-finned fish | *Salmo salar* | Atlantic salmon | GCF_905237065.1 |
| Ray-finned fish | *Oryzias latipes* | Medaka | GCF_002234675.1 |
| Ray-finned fish | *Astyanax mexicanus* | Mexican tetra | GCF_023375975.1 |
| Ray-finned fish | *Oreochromis niloticus* | Nile tilapia | GCF_001858045.2 |
| Lobe-finned fish | *Latimeria chalumnae* | West Indian Ocean coelacanth | GCF_000225785.1 |
| Amphibia | *Xenopus tropicalis* | Western clawed frog | GCF_000004195.4 |
| Amphibia | *Xenopus laevis* | African clawed frog | GCF_017654675.1 |
| Sauropsida | *Gallus gallus* | Chicken | GCF_016699485.2 |
| Sauropsida | *Anolis carolinensis* | Green anole | GCF_000090745.1 |
| Sauropsida | *Taeniopygia guttata* | Sunda zebra finch | GCF_003957565.2 |
| Sauropsida | *Gopherus evgoodei* | Goode's thornscrub tortoise | GCF_007399415.2 |
| Sauropsida | *Melopsittacus undulatus* | Common parakeet | GCF_012275295.1 |
| Mammalia | *Ornithorhynchus anatinus* | Platypus | GCF_004115215.2 |
| Mammalia | *Monodelphis domestica* | Opossum | GCF_027887165.1 |
| Mammalia | *Canis lupus familiaris* | Dog | GCF_000002285.5 |
| Mammalia | *Ovis aries* | Sheep | GCF_016772045.2 |
| Mammalia | *Mus musculus* | Mouse | GCF_000001635.27 |
| Mammalia | *Pan paniscus* | Bonobo | GCF_029289425.1 |
| Mammalia | *Homo sapiens* | Human | GCF_000001405.40 |
| Outgroup | *Trichoplax adhaerens* |  | GCF_000150275.1 |
| Outgroup | *Mizuhopecten yessoensis* | Yesso scallop | GCF_002113885.1 |
| Outgroup | *Strongylocentrotus purpuratus* | Purple sea urchin | GCF_000002235.5 |
| Outgroup | *Drosophila melanogaster* |  | GCF_000001215.4 |
| Outgroup | *Caenorhabditis elegans* |  | GCF_000002985.6 |

**Table S2**. Ohnolog status based on three publications.

|  | If ohnolog from Vance and McLysaght 2023 [37] | | |
| --- | --- | --- | --- |
| ATG_gene | Makino and McLysaght 2010 [35] | Singh and Isambert 2020 [36] | Nakatani et al. 2021 (analyzed by Vance and McLysaght 2023) [31,37] |
| *ULK1* | T | T | F |
| *ULK2* |  |  |  |
| *ATG9A* | T | T | F |
| *ATG9B* |  |  |  |
| *BECN1* | F | F | F |
| *BECN2* |  |  |  |
| *ATG2A* | T | T | F |
| *ATG2B* |  |  |  |
| *WIPI1* | T | T | F |
| *WIPI2* |  |  |  |
| *GABARAP* | T | F | F |
| *GABARAPL1* |  |  |  |
| *LC3A* | T | T | F |
| *LC3B* |  |  |  |
| *ATG4A* | F | T | F |
| *ATG4B* |  |  |  |
| *ATG4C* | T | T | F |
| *ATG4D* |  |  |  |
| *ATG16L1* | T | T | F |
| *ATG16L2* |  |  |  |

**Table S3**. List of RNA-seq samples in the 14 species used in the gene expression evolution analysis.

| Species | Genome id | SRA id | Tissue name | Tissue category | Citation of data source |
| --- | --- | --- | --- | --- | --- |
| *Branchiostoma lanceolatum* | GCA_927797965.1 | SRR6246036 | Neural tube | Brain | Marlétaz et al. 2018 [43] |
| *Branchiostoma lanceolatum* | GCA_927797965.1 | SRR6246037 | Neural tube | Brain |  |
| *Branchiostoma lanceolatum* | GCA_927797965.1 | SRR6246028 | Gut | Intestine |  |
| *Branchiostoma lanceolatum* | GCA_927797965.1 | SRR6246029 | Gut | Intestine |  |
| *Branchiostoma lanceolatum* | GCA_927797965.1 | SRR6246030 | Hepatic diverticulum | Liver |  |
| *Branchiostoma lanceolatum* | GCA_927797965.1 | SRR6246031 | Hepatic diverticulum | Liver |  |
| *Branchiostoma lanceolatum* | GCA_927797965.1 | SRR6246025 | Female gonads | Ovary |  |
| *Branchiostoma lanceolatum* | GCA_927797965.1 | SRR6246032 | Male gonads | Testis |  |
| *Branchiostoma lanceolatum* | GCA_927797965.1 | SRR6246033 | Male gonads | Testis |  |
| *Eptatretus burgeri* | GCA_900186335.2 | ERR2061179 | Blood | Blood | Yu et al. 2024 [34] |
| *Eptatretus burgeri* | GCA_900186335.2 | ERR2061180 | Blood | Blood |  |
| *Eptatretus burgeri* | GCA_900186335.2 | ERR2061163 | Brain | Brain |  |
| *Eptatretus burgeri* | GCA_900186335.2 | ERR2061164 | Brain | Brain |  |
| *Eptatretus burgeri* | GCA_900186335.2 | ERR2061177 | Gill | Gill |  |
| *Eptatretus burgeri* | GCA_900186335.2 | ERR2061178 | Gill | Gill |  |
| *Eptatretus burgeri* | GCA_900186335.2 | ERR2061167 | Heart | Heart |  |
| *Eptatretus burgeri* | GCA_900186335.2 | ERR2061168 | Heart | Heart |  |
| *Eptatretus burgeri* | GCA_900186335.2 | ERR2061173 | Intestine | Intestine |  |
| *Eptatretus burgeri* | GCA_900186335.2 | ERR2061174 | Intestine | Intestine |  |
| *Eptatretus burgeri* | GCA_900186335.2 | ERR2061169 | Kidney | Kidney |  |
| *Eptatretus burgeri* | GCA_900186335.2 | ERR2061170 | Kidney | Kidney |  |
| *Eptatretus burgeri* | GCA_900186335.2 | ERR2061175 | Liver | Liver |  |
| *Eptatretus burgeri* | GCA_900186335.2 | ERR2061176 | Liver | Liver |  |
| *Eptatretus burgeri* | GCA_900186335.2 | ERR2061171 | Skeletal muscle | Muscle |  |
| *Eptatretus burgeri* | GCA_900186335.2 | ERR2061172 | Skeletal muscle | Muscle |  |
| *Eptatretus burgeri* | GCA_900186335.2 | ERR2061165 | Testis | Testis |  |
| *Eptatretus burgeri* | GCA_900186335.2 | ERR2061166 | Testis | Testis |  |
| *Leucoraja erinacea* | GCF_028641065.1 | SRR24105886 | Midbrain | Brain | Marlétaz et al. 2023 [44] |
| *Leucoraja erinacea* | GCF_028641065.1 | SRR24105887 | Midbrain | Brain |  |
| *Leucoraja erinacea* | GCF_028641065.1 | SRR24105894 | Hindbrain | Brain |  |
| *Leucoraja erinacea* | GCF_028641065.1 | SRR24105895 | Hindbrain | Brain |  |
| *Leucoraja erinacea* | GCF_028641065.1 | SRR24105900 | Forebrain | Brain |  |
| *Leucoraja erinacea* | GCF_028641065.1 | SRR24105901 | Forebrain | Brain |  |
| *Leucoraja erinacea* | GCF_028641065.1 | SRR24105902 | Cerebellum | Cerebellum |  |
| *Leucoraja erinacea* | GCF_028641065.1 | SRR24105903 | Cerebellum | Cerebellum |  |
| *Leucoraja erinacea* | GCF_028641065.1 | SRR24105898 | Gill | Gill |  |
| *Leucoraja erinacea* | GCF_028641065.1 | SRR24105899 | Gill | Gill |  |
| *Leucoraja erinacea* | GCF_028641065.1 | SRR24105896 | Heart | Heart |  |
| *Leucoraja erinacea* | GCF_028641065.1 | SRR24105897 | Heart | Heart |  |
| *Leucoraja erinacea* | GCF_028641065.1 | SRR24105892 | Intestine | Intestine |  |
| *Leucoraja erinacea* | GCF_028641065.1 | SRR24105893 | Intestine | Intestine |  |
| *Leucoraja erinacea* | GCF_028641065.1 | SRR24105890 | Kidney | Kidney |  |
| *Leucoraja erinacea* | GCF_028641065.1 | SRR24105891 | Kidney | Kidney |  |
| *Leucoraja erinacea* | GCF_028641065.1 | SRR24105888 | Liver | Liver |  |
| *Leucoraja erinacea* | GCF_028641065.1 | SRR24105889 | Liver | Liver |  |
| *Leucoraja erinacea* | GCF_028641065.1 | SRR24105884 | Muscle | Muscle |  |
| *Leucoraja erinacea* | GCF_028641065.1 | SRR24105885 | Muscle | Muscle |  |
| *Leucoraja erinacea* | GCF_028641065.1 | SRR24105882 | Ovary | Ovary |  |
| *Leucoraja erinacea* | GCF_028641065.1 | SRR24105883 | Ovary | Ovary |  |
| *Leucoraja erinacea* | GCF_028641065.1 | SRR24105880 | Pancreas | Pancreas |  |
| *Leucoraja erinacea* | GCF_028641065.1 | SRR24105881 | Pancreas | Pancreas |  |
| *Leucoraja erinacea* | GCF_028641065.1 | SRR24105878 | Testis | Testis |  |
| *Leucoraja erinacea* | GCF_028641065.1 | SRR24105879 | Testis | Testis |  |
| *Danio rerio* | GCF_000002035.6 | SRR1524238 | Brain | Brain | Pasquier et al. 2016 [45] |
| *Danio rerio* | GCF_000002035.6 | SRR1524240 | Heart | Heart |  |
| *Danio rerio* | GCF_000002035.6 | SRR1524245 | Intestine | Intestine |  |
| *Danio rerio* | GCF_000002035.6 | SRR1524243 | Kidney | Kidney |  |
| *Danio rerio* | GCF_000002035.6 | SRR1524242 | Liver | Liver |  |
| *Danio rerio* | GCF_000002035.6 | SRR1524248 | Ovary | Ovary |  |
| *Danio rerio* | GCF_000002035.6 | SRR1524249 | Testis | Testis |  |
| *Oryzias latipes* | GCF_002234675.1 | SRR1524271 | Brain | Brain |  |
| *Oryzias latipes* | GCF_002234675.1 | SRR1524273 | Heart | Heart |  |
| *Oryzias latipes* | GCF_002234675.1 | SRR1524278 | Intestine | Intestine |  |
| *Oryzias latipes* | GCF_002234675.1 | SRR1524276 | Kidney | Kidney |  |
| *Oryzias latipes* | GCF_002234675.1 | SRR1524275 | Liver | Liver |  |
| *Oryzias latipes* | GCF_002234675.1 | SRR1524280 | Ovary | Ovary |  |
| *Oryzias latipes* | GCF_002234675.1 | SRR1524281 | Testis | Testis |  |
| *Xenopus tropicalis* | GCF_000004195.4 | SRR649391 | Brain | Brain | Necsulea et al. 2014 [46] |
| *Xenopus tropicalis* | GCF_000004195.4 | SRR649392 | Brain | Brain |  |
| *Xenopus tropicalis* | GCF_000004195.4 | SRR649393 | Heart | Heart |  |
| *Xenopus tropicalis* | GCF_000004195.4 | SRR649394 | Heart | Heart |  |
| *Xenopus tropicalis* | GCF_000004195.4 | SRR649395 | Kidney | Kidney |  |
| *Xenopus tropicalis* | GCF_000004195.4 | SRR649396 | Kidney | Kidney |  |
| *Xenopus tropicalis* | GCF_000004195.4 | SRR649397 | Liver | Liver |  |
| *Xenopus tropicalis* | GCF_000004195.4 | SRR649398 | Liver | Liver |  |
| *Xenopus tropicalis* | GCF_000004195.4 | SRR943352 | Ovary | Ovary |  |
| *Xenopus tropicalis* | GCF_000004195.4 | SRR649400 | Ovary | Ovary |  |
| *Xenopus tropicalis* | GCF_000004195.4 | SRR943353 | Testis | Testis |  |
| *Xenopus tropicalis* | GCF_000004195.4 | SRR649399 | Testis | Testis |  |
| *Gallus gallus* | GCF_016699485.2 | SRR649385 | Brain | Brain | Brawand et al. 2011 [47],  Necsulea et al. 2014 [46] |
| *Gallus gallus* | GCF_016699485.2 | SRR306710 | Brain | Brain |  |
| *Gallus gallus* | GCF_016699485.2 | SRR306711 | Brain | Brain |  |
| *Gallus gallus* | GCF_016699485.2 | SRR306712 | Cerebellum | Cerebellum |  |
| *Gallus gallus* | GCF_016699485.2 | SRR306713 | Cerebellum | Cerebellum |  |
| *Gallus gallus* | GCF_016699485.2 | SRR306714 | Heart | Heart |  |
| *Gallus gallus* | GCF_016699485.2 | SRR306715 | Heart | Heart |  |
| *Gallus gallus* | GCF_016699485.2 | SRR306716 | Kidney | Kidney |  |
| *Gallus gallus* | GCF_016699485.2 | SRR306717 | Kidney | Kidney |  |
| *Gallus gallus* | GCF_016699485.2 | SRR306718 | Liver | Liver |  |
| *Gallus gallus* | GCF_016699485.2 | SRR306719 | Liver | Liver |  |
| *Gallus gallus* | GCF_016699485.2 | SRR306720 | Liver | Liver |  |
| *Gallus gallus* | GCF_016699485.2 | SRR943351 | Ovary | Ovary |  |
| *Gallus gallus* | GCF_016699485.2 | SRR649386 | Ovary | Ovary |  |
| *Gallus gallus* | GCF_016699485.2 | SRR649387 | Ovary | Ovary |  |
| *Gallus gallus* | GCF_016699485.2 | SRR649388 | Ovary | Ovary |  |
| *Gallus gallus* | GCF_016699485.2 | SRR649389 | Testis | Testis |  |
| *Gallus gallus* | GCF_016699485.2 | SRR649390 | Testis | Testis |  |
| *Gallus gallus* | GCF_016699485.2 | SRR306721 | Testis | Testis |  |
| *Gallus gallus* | GCF_016699485.2 | SRR306722 | Testis | Testis |  |
| *Gallus gallus* | GCF_016699485.2 | SRR306723 | Testis | Testis |  |
| *Ornithorhynchus anatinus* | GCF_004115215.2 | SRR649381 | Brain | Brain |  |
| *Ornithorhynchus anatinus* | GCF_004115215.2 | SRR306724 | Brain | Brain |  |
| *Ornithorhynchus anatinus* | GCF_004115215.2 | SRR306725 | Brain | Brain |  |
| *Ornithorhynchus anatinus* | GCF_004115215.2 | SRR306726 | Brain | Brain |  |
| *Ornithorhynchus anatinus* | GCF_004115215.2 | SRR306727 | Brain | Brain |  |
| *Ornithorhynchus anatinus* | GCF_004115215.2 | SRR306728 | Cerebellum | Cerebellum |  |
| *Ornithorhynchus anatinus* | GCF_004115215.2 | SRR306729 | Cerebellum | Cerebellum |  |
| *Ornithorhynchus anatinus* | GCF_004115215.2 | SRR306730 | Heart | Heart |  |
| *Ornithorhynchus anatinus* | GCF_004115215.2 | SRR306731 | Heart | Heart |  |
| *Ornithorhynchus anatinus* | GCF_004115215.2 | SRR306732 | Kidney | Kidney |  |
| *Ornithorhynchus anatinus* | GCF_004115215.2 | SRR306734 | Kidney | Kidney |  |
| *Ornithorhynchus anatinus* | GCF_004115215.2 | SRR306735 | Liver | Liver |  |
| *Ornithorhynchus anatinus* | GCF_004115215.2 | SRR306736 | Liver | Liver |  |
| *Ornithorhynchus anatinus* | GCF_004115215.2 | SRR306737 | Liver | Liver |  |
| *Ornithorhynchus anatinus* | GCF_004115215.2 | SRR306738 | Liver | Liver |  |
| *Ornithorhynchus anatinus* | GCF_004115215.2 | SRR943349 | Ovary | Ovary |  |
| *Ornithorhynchus anatinus* | GCF_004115215.2 | SRR943350 | Ovary | Ovary |  |
| *Ornithorhynchus anatinus* | GCF_004115215.2 | SRR649382 | Ovary | Ovary |  |
| *Ornithorhynchus anatinus* | GCF_004115215.2 | SRR649383 | Testis | Testis |  |
| *Ornithorhynchus anatinus* | GCF_004115215.2 | SRR649384 | Testis | Testis |  |
| *Ornithorhynchus anatinus* | GCF_004115215.2 | SRR306739 | Testis | Testis |  |
| *Ornithorhynchus anatinus* | GCF_004115215.2 | SRR306740 | Testis | Testis |  |
| *Ornithorhynchus anatinus* | GCF_004115215.2 | SRR306741 | Testis | Testis |  |
| *Monodelphis domestica* | GCF_027887165.1 | SRR649376 | Brain | Brain | Brawand et al. 2011 [47],  Necsulea et al. 2014 [46],  Armstrong et al. 2017 [48] |
| *Monodelphis domestica* | GCF_027887165.1 | SRR306742 | Brain | Brain |  |
| *Monodelphis domestica* | GCF_027887165.1 | SRR306743 | Brain | Brain |  |
| *Monodelphis domestica* | GCF_027887165.1 | SRR306744 | Brain | Brain |  |
| *Monodelphis domestica* | GCF_027887165.1 | SRR306745 | Cerebellum | Cerebellum |  |
| *Monodelphis domestica* | GCF_027887165.1 | SRR306746 | Cerebellum | Cerebellum |  |
| *Monodelphis domestica* | GCF_027887165.1 | SRR306747 | Heart | Heart |  |
| *Monodelphis domestica* | GCF_027887165.1 | SRR306748 | Heart | Heart |  |
| *Monodelphis domestica* | GCF_027887165.1 | SRR306749 | Heart | Heart |  |
| *Monodelphis domestica* | GCF_027887165.1 | SRR306750 | Heart | Heart |  |
| *Monodelphis domestica* | GCF_027887165.1 | SRR306751 | Kidney | Kidney |  |
| *Monodelphis domestica* | GCF_027887165.1 | SRR306752 | Kidney | Kidney |  |
| *Monodelphis domestica* | GCF_027887165.1 | SRR306753 | Liver | Liver |  |
| *Monodelphis domestica* | GCF_027887165.1 | SRR306754 | Liver | Liver |  |
| *Monodelphis domestica* | GCF_027887165.1 | SRR943346 | Ovary | Ovary |  |
| *Monodelphis domestica* | GCF_027887165.1 | SRR649377 | Ovary | Ovary |  |
| *Monodelphis domestica* | GCF_027887165.1 | SRR3222432 | Placenta | Placenta |  |
| *Monodelphis domestica* | GCF_027887165.1 | SRR649379 | Testis | Testis |  |
| *Monodelphis domestica* | GCF_027887165.1 | SRR649380 | Testis | Testis |  |
| *Monodelphis domestica* | GCF_027887165.1 | SRR306755 | Testis | Testis |  |
| *Monodelphis domestica* | GCF_027887165.1 | SRR306756 | Testis | Testis |  |
| *Ovis aries* | GCF_016772045.2 | ERR489148 | Cerebellum | Cerebellum | University of Edinburgh sheep gene expression atlas,  Archibald et al. 2010 [49] |
| *Ovis aries* | GCF_016772045.2 | ERR489149 | Cerebellum | Cerebellum |  |
| *Ovis aries* | GCF_016772045.2 | ERR489150 | Cerebellum | Cerebellum |  |
| *Ovis aries* | GCF_016772045.2 | ERR489151 | Cerebellum | Cerebellum |  |
| *Ovis aries* | GCF_016772045.2 | ERR489216 | Brain cerebellum | Cerebellum |  |
| *Ovis aries* | GCF_016772045.2 | ERR489217 | Brain cerebellum | Cerebellum |  |
| *Ovis aries* | GCF_016772045.2 | ERR489219 | Brain cerebrum | Brain |  |
| *Ovis aries* | GCF_016772045.2 | ERR489258 | Ventricle | Heart |  |
| *Ovis aries* | GCF_016772045.2 | ERR489259 | Ventricle | Heart |  |
| *Ovis aries* | GCF_016772045.2 | ERR489268 | Heart vertricle | Heart |  |
| *Ovis aries* | GCF_016772045.2 | ERR489269 | Heart vertricle | Heart |  |
| *Ovis aries* | GCF_016772045.2 | ERR489168 | Colon | Intestine |  |
| *Ovis aries* | GCF_016772045.2 | ERR489169 | Colon | Intestine |  |
| *Ovis aries* | GCF_016772045.2 | ERR489170 | Colon | Intestine |  |
| *Ovis aries* | GCF_016772045.2 | ERR489171 | Colon | Intestine |  |
| *Ovis aries* | GCF_016772045.2 | ERR489172 | Colon | Intestine |  |
| *Ovis aries* | GCF_016772045.2 | ERR489173 | Colon | Intestine |  |
| *Ovis aries* | GCF_016772045.2 | ERR489212 | Colon | Intestine |  |
| *Ovis aries* | GCF_016772045.2 | ERR489213 | Colon | Intestine |  |
| *Ovis aries* | GCF_016772045.2 | ERR489260 | Kidney cortex | Kidney |  |
| *Ovis aries* | GCF_016772045.2 | ERR489261 | Kidney cortex | Kidney |  |
| *Ovis aries* | GCF_016772045.2 | ERR489262 | Kidney medulla | Kidney |  |
| *Ovis aries* | GCF_016772045.2 | ERR489263 | Kidney medulla | Kidney |  |
| *Ovis aries* | GCF_016772045.2 | ERR489271 | Kidney medulla | Kidney |  |
| *Ovis aries* | GCF_016772045.2 | ERR489264 | Liver | Liver |  |
| *Ovis aries* | GCF_016772045.2 | ERR489265 | Liver | Liver |  |
| *Ovis aries* | GCF_016772045.2 | ERR489272 | Liver | Liver |  |
| *Ovis aries* | GCF_016772045.2 | ERR489273 | Liver | Liver |  |
| *Ovis aries* | GCF_016772045.2 | ERR489140 | Ovary | Ovary |  |
| *Ovis aries* | GCF_016772045.2 | ERR489141 | Ovary | Ovary |  |
| *Ovis aries* | GCF_016772045.2 | ERR489144 | Placenta | Placenta |  |
| *Ovis aries* | GCF_016772045.2 | ERR489145 | Placenta | Placenta |  |
| *Ovis aries* | GCF_016772045.2 | ERR489222 | Testes | Testis |  |
| *Ovis aries* | GCF_016772045.2 | ERR489223 | Testes | Testis |  |
| *Canis lupus familiaris* | GCF_000002285.5 | SRR8996954 | Occipital cortex | Brain | Broad Institute,  Armstrong et al. 2017 [48] |
| *Canis lupus familiaris* | GCF_000002285.5 | SRR8996959 | Frontal cortex | Brain |  |
| *Canis lupus familiaris* | GCF_000002285.5 | SRR8996997 | Frontal cortex | Brain |  |
| *Canis lupus familiaris* | GCF_000002285.5 | SRR8996998 | Occipital cortex | Brain |  |
| *Canis lupus familiaris* | GCF_000002285.5 | SRR8997012 | Occipital cortex | Brain |  |
| *Canis lupus familiaris* | GCF_000002285.5 | SRR8997021 | Frontal cortex | Brain |  |
| *Canis lupus familiaris* | GCF_000002285.5 | SRR8997030 | Occipital cortex | Brain |  |
| *Canis lupus familiaris* | GCF_000002285.5 | SRR8997033 | Frontal cortex | Brain |  |
| *Canis lupus familiaris* | GCF_000002285.5 | SRR5889305 | Frontal cortex | Brain |  |
| *Canis lupus familiaris* | GCF_000002285.5 | SRR5889306 | Occipital cortex | Brain |  |
| *Canis lupus familiaris* | GCF_000002285.5 | SRR8996999 | Cerebellum | Cerebellum |  |
| *Canis lupus familiaris* | GCF_000002285.5 | SRR8997015 | Cerebellum | Cerebellum |  |
| *Canis lupus familiaris* | GCF_000002285.5 | SRR8997035 | Cerebellum | Cerebellum |  |
| *Canis lupus familiaris* | GCF_000002285.5 | SRR5889308 | Cerebellum | Cerebellum |  |
| *Canis lupus familiaris* | GCF_000002285.5 | SRR8996963 | Left ventricle | Heart |  |
| *Canis lupus familiaris* | GCF_000002285.5 | SRR8996964 | Left atrium | Heart |  |
| *Canis lupus familiaris* | GCF_000002285.5 | SRR8996969 | Right ventricle | Heart |  |
| *Canis lupus familiaris* | GCF_000002285.5 | SRR8996973 | Right ventricle | Heart |  |
| *Canis lupus familiaris* | GCF_000002285.5 | SRR8996974 | Right atrium | Heart |  |
| *Canis lupus familiaris* | GCF_000002285.5 | SRR8996975 | Left atrium | Heart |  |
| *Canis lupus familiaris* | GCF_000002285.5 | SRR8996978 | Left ventricle | Heart |  |
| *Canis lupus familiaris* | GCF_000002285.5 | SRR8996991 | Right ventricle | Heart |  |
| *Canis lupus familiaris* | GCF_000002285.5 | SRR8996992 | Right atrium | Heart |  |
| *Canis lupus familiaris* | GCF_000002285.5 | SRR8997007 | Left atrium | Heart |  |
| *Canis lupus familiaris* | GCF_000002285.5 | SRR8997008 | Left ventricle | Heart |  |
| *Canis lupus familiaris* | GCF_000002285.5 | SRR8997025 | Right ventricle | Heart |  |
| *Canis lupus familiaris* | GCF_000002285.5 | SRR8997028 | Right atrium | Heart |  |
| *Canis lupus familiaris* | GCF_000002285.5 | SRR8997037 | Left ventricle | Heart |  |
| *Canis lupus familiaris* | GCF_000002285.5 | SRR8997038 | Left atrium | Heart |  |
| *Canis lupus familiaris* | GCF_000002285.5 | SRR5889335 | Left atrium | Heart |  |
| *Canis lupus familiaris* | GCF_000002285.5 | SRR5889336 | Left ventricle | Heart |  |
| *Canis lupus familiaris* | GCF_000002285.5 | SRR5889342 | Right atrium | Heart |  |
| *Canis lupus familiaris* | GCF_000002285.5 | SRR5889343 | Right ventricle | Heart |  |
| *Canis lupus familiaris* | GCF_000002285.5 | SRR8996987 | Small intestine | Intestine |  |
| *Canis lupus familiaris* | GCF_000002285.5 | SRR8997000 | Colon | Intestine |  |
| *Canis lupus familiaris* | GCF_000002285.5 | SRR8997022 | Colon | Intestine |  |
| *Canis lupus familiaris* | GCF_000002285.5 | SRR8997031 | Small intestine | Intestine |  |
| *Canis lupus familiaris* | GCF_000002285.5 | SRR8997034 | Colon | Intestine |  |
| *Canis lupus familiaris* | GCF_000002285.5 | SRR8997056 | Small intestine | Intestine |  |
| *Canis lupus familiaris* | GCF_000002285.5 | SRR5889307 | Colon | Intestine |  |
| *Canis lupus familiaris* | GCF_000002285.5 | SRR5889348 | Small intestine | Intestine |  |
| *Canis lupus familiaris* | GCF_000002285.5 | SRR8996953 | Kidney cortex | Kidney |  |
| *Canis lupus familiaris* | GCF_000002285.5 | SRR8996976 | Kidney medulla | Kidney |  |
| *Canis lupus familiaris* | GCF_000002285.5 | SRR8997001 | Kidney cortex | Kidney |  |
| *Canis lupus familiaris* | GCF_000002285.5 | SRR8997002 | Kidney medulla | Kidney |  |
| *Canis lupus familiaris* | GCF_000002285.5 | SRR8997005 | Kidney cortex | Kidney |  |
| *Canis lupus familiaris* | GCF_000002285.5 | SRR8997006 | Kidney medulla | Kidney |  |
| *Canis lupus familiaris* | GCF_000002285.5 | SRR8997039 | Kidney medulla | Kidney |  |
| *Canis lupus familiaris* | GCF_000002285.5 | SRR8997040 | Kidney cortex | Kidney |  |
| *Canis lupus familiaris* | GCF_000002285.5 | SRR5889313 | Kidney medulla | Kidney |  |
| *Canis lupus familiaris* | GCF_000002285.5 | SRR5889314 | Kidney cortex | Kidney |  |
| *Canis lupus familiaris* | GCF_000002285.5 | SRR8996966 | Liver | Liver |  |
| *Canis lupus familiaris* | GCF_000002285.5 | SRR8996977 | Liver | Liver |  |
| *Canis lupus familiaris* | GCF_000002285.5 | SRR8997009 | Liver | Liver |  |
| *Canis lupus familiaris* | GCF_000002285.5 | SRR8997042 | Liver | Liver |  |
| *Canis lupus familiaris* | GCF_000002285.5 | SRR5889337 | Liver | Liver |  |
| *Canis lupus familiaris* | GCF_000002285.5 | SRR8996971 | Skeletal muscle | Muscle |  |
| *Canis lupus familiaris* | GCF_000002285.5 | SRR8996989 | Skeletal muscle | Muscle |  |
| *Canis lupus familiaris* | GCF_000002285.5 | SRR8997023 | Skeletal muscle | Muscle |  |
| *Canis lupus familiaris* | GCF_000002285.5 | SRR8997054 | Skeletal muscle | Muscle |  |
| *Canis lupus familiaris* | GCF_000002285.5 | SRR5889346 | Skeletal muscle | Muscle |  |
| *Canis lupus familiaris* | GCF_000002285.5 | SRR8996967 | Pancreas | Pancreas |  |
| *Canis lupus familiaris* | GCF_000002285.5 | SRR8996982 | Pancreas | Pancreas |  |
| *Canis lupus familiaris* | GCF_000002285.5 | SRR8997003 | Pancreas | Pancreas |  |
| *Canis lupus familiaris* | GCF_000002285.5 | SRR8997027 | Pancreas | Pancreas |  |
| *Canis lupus familiaris* | GCF_000002285.5 | SRR5889340 | Pancreas | Pancreas |  |
| *Canis lupus familiaris* | GCF_000002285.5 | SRR3222429 | Placenta | Placenta |  |
| *Mus musculus* | GCF_000001635.27 | SRR649371 | Brain | Brain | Brawand et al. 2011 [47],  Necsulea et al. 2014 [46],  Wang et al. 2011 [50] |
| *Mus musculus* | GCF_000001635.27 | SRR306757 | Brain | Brain |  |
| *Mus musculus* | GCF_000001635.27 | SRR306758 | Brain | Brain |  |
| *Mus musculus* | GCF_000001635.27 | SRR306759 | Brain | Brain |  |
| *Mus musculus* | GCF_000001635.27 | SRR306760 | Brain | Brain |  |
| *Mus musculus* | GCF_000001635.27 | SRR306761 | Brain | Brain |  |
| *Mus musculus* | GCF_000001635.27 | SRR306762 | Brain | Brain |  |
| *Mus musculus* | GCF_000001635.27 | SRR306763 | Cerebellum | Cerebellum |  |
| *Mus musculus* | GCF_000001635.27 | SRR306764 | Cerebellum | Cerebellum |  |
| *Mus musculus* | GCF_000001635.27 | SRR306765 | Cerebellum | Cerebellum |  |
| *Mus musculus* | GCF_000001635.27 | SRR306766 | Heart | Heart |  |
| *Mus musculus* | GCF_000001635.27 | SRR306767 | Heart | Heart |  |
| *Mus musculus* | GCF_000001635.27 | SRR306768 | Heart | Heart |  |
| *Mus musculus* | GCF_000001635.27 | SRR306769 | Kidney | Kidney |  |
| *Mus musculus* | GCF_000001635.27 | SRR306770 | Kidney | Kidney |  |
| *Mus musculus* | GCF_000001635.27 | SRR306771 | Kidney | Kidney |  |
| *Mus musculus* | GCF_000001635.27 | SRR306772 | Liver | Liver |  |
| *Mus musculus* | GCF_000001635.27 | SRR306773 | Liver | Liver |  |
| *Mus musculus* | GCF_000001635.27 | SRR306774 | Liver | Liver |  |
| *Mus musculus* | GCF_000001635.27 | SRR943342 | Ovary | Ovary |  |
| *Mus musculus* | GCF_000001635.27 | SRR943343 | Ovary | Ovary |  |
| *Mus musculus* | GCF_000001635.27 | SRR649372 | Ovary | Ovary |  |
| *Mus musculus* | GCF_000001635.27 | SRR243586 | Placenta | Placenta |  |
| *Mus musculus* | GCF_000001635.27 | SRR243587 | Placenta | Placenta |  |
| *Mus musculus* | GCF_000001635.27 | SRR243588 | Placenta | Placenta |  |
| *Mus musculus* | GCF_000001635.27 | SRR243589 | Placenta | Placenta |  |
| *Mus musculus* | GCF_000001635.27 | SRR243591 | Placenta | Placenta |  |
| *Mus musculus* | GCF_000001635.27 | SRR243592 | Placenta | Placenta |  |
| *Mus musculus* | GCF_000001635.27 | SRR243594 | Placenta | Placenta |  |
| *Mus musculus* | GCF_000001635.27 | SRR243595 | Placenta | Placenta |  |
| *Mus musculus* | GCF_000001635.27 | SRR243596 | Placenta | Placenta |  |
| *Mus musculus* | GCF_000001635.27 | SRR243597 | Placenta | Placenta |  |
| *Mus musculus* | GCF_000001635.27 | SRR243598 | Placenta | Placenta |  |
| *Mus musculus* | GCF_000001635.27 | SRR243599 | Placenta | Placenta |  |
| *Mus musculus* | GCF_000001635.27 | SRR243600 | Placenta | Placenta |  |
| *Mus musculus* | GCF_000001635.27 | SRR243601 | Placenta | Placenta |  |
| *Mus musculus* | GCF_000001635.27 | SRR243602 | Placenta | Placenta |  |
| *Mus musculus* | GCF_000001635.27 | SRR649375 | Testis | Testis |  |
| *Mus musculus* | GCF_000001635.27 | SRR306775 | Testis | Testis |  |
| *Mus musculus* | GCF_000001635.27 | SRR306776 | Testis | Testis |  |
| *Pan paniscus* | GCF_029289425.1 | SRR306826 | Brain prefrontal cortex | Brain | Brawand et al. 2011 [47],  Necsulea et al. 2014 [46],  Armstrong et al. 2017 [48] |
| *Pan paniscus* | GCF_029289425.1 | SRR306827 | Brain prefrontal cortex | Brain |  |
| *Pan paniscus* | GCF_029289425.1 | SRR306828 | Brain prefrontal cortex | Brain |  |
| *Pan paniscus* | GCF_029289425.1 | SRR306829 | Cerebellum | Cerebellum |  |
| *Pan paniscus* | GCF_029289425.1 | SRR306830 | Cerebellum | Cerebellum |  |
| *Pan paniscus* | GCF_029289425.1 | SRR306831 | Heart | Heart |  |
| *Pan paniscus* | GCF_029289425.1 | SRR306832 | Heart | Heart |  |
| *Pan paniscus* | GCF_029289425.1 | SRR306833 | Kidney | Kidney |  |
| *Pan paniscus* | GCF_029289425.1 | SRR306834 | Kidney | Kidney |  |
| *Pan paniscus* | GCF_029289425.1 | SRR306835 | Liver | Liver |  |
| *Pan paniscus* | GCF_029289425.1 | SRR306836 | Liver | Liver |  |
| *Pan paniscus* | GCF_029289425.1 | SRR3222427 | Placenta | Placenta |  |
| *Pan paniscus* | GCF_029289425.1 | SRR306837 | Testis | Testis |  |
| *Homo sapiens* | GCF_000001405.40 | SRR943339 | Brain frontal cortex | Brain | Brawand et al. 2011 [47],  Necsulea et al. 2014 [46],  Hughes et al. 2015 [51] |
| *Homo sapiens* | GCF_000001405.40 | SRR943357 | Brain frontal cortex | Brain |  |
| *Homo sapiens* | GCF_000001405.40 | SRR943358 | Brain frontal cortex | Brain |  |
| *Homo sapiens* | GCF_000001405.40 | SRR649360 | Brain frontal cortex | Brain |  |
| *Homo sapiens* | GCF_000001405.40 | SRR649361 | Brain frontal cortex | Brain |  |
| *Homo sapiens* | GCF_000001405.40 | SRR306838 | Brain frontal cortex | Brain |  |
| *Homo sapiens* | GCF_000001405.40 | SRR306839 | Brain frontal cortex | Brain |  |
| *Homo sapiens* | GCF_000001405.40 | SRR306840 | Brain prefrontal cortex | Brain |  |
| *Homo sapiens* | GCF_000001405.40 | SRR306841 | Brain prefrontal cortex | Brain |  |
| *Homo sapiens* | GCF_000001405.40 | SRR306842 | Brain prefrontal cortex | Brain |  |
| *Homo sapiens* | GCF_000001405.40 | SRR306843 | Brain temporal lobe | Brain |  |
| *Homo sapiens* | GCF_000001405.40 | SRR306844 | Cerebellum | Cerebellum |  |
| *Homo sapiens* | GCF_000001405.40 | SRR306845 | Cerebellum | Cerebellum |  |
| *Homo sapiens* | GCF_000001405.40 | SRR306846 | Cerebellum | Cerebellum |  |
| *Homo sapiens* | GCF_000001405.40 | SRR306847 | Heart | Heart |  |
| *Homo sapiens* | GCF_000001405.40 | SRR306848 | Heart | Heart |  |
| *Homo sapiens* | GCF_000001405.40 | SRR306849 | Heart | Heart |  |
| *Homo sapiens* | GCF_000001405.40 | SRR306850 | Heart | Heart |  |
| *Homo sapiens* | GCF_000001405.40 | SRR306851 | Kidney | Kidney |  |
| *Homo sapiens* | GCF_000001405.40 | SRR306852 | Kidney | Kidney |  |
| *Homo sapiens* | GCF_000001405.40 | SRR306853 | Kidney | Kidney |  |
| *Homo sapiens* | GCF_000001405.40 | SRR306854 | Liver | Liver |  |
| *Homo sapiens* | GCF_000001405.40 | SRR306855 | Liver | Liver |  |
| *Homo sapiens* | GCF_000001405.40 | SRR306856 | Liver | Liver |  |
| *Homo sapiens* | GCF_000001405.40 | SRR943341 | Ovary | Ovary |  |
| *Homo sapiens* | GCF_000001405.40 | SRR649364 | Ovary | Ovary |  |
| *Homo sapiens* | GCF_000001405.40 | SRR1850939 | placenta | Placenta |  |
| *Homo sapiens* | GCF_000001405.40 | SRR1850941 | placenta | Placenta |  |
| *Homo sapiens* | GCF_000001405.40 | SRR1850944 | placenta | Placenta |  |
| *Homo sapiens* | GCF_000001405.40 | SRR1850952 | placenta | Placenta |  |
| *Homo sapiens* | GCF_000001405.40 | SRR1850956 | placenta | Placenta |  |
| *Homo sapiens* | GCF_000001405.40 | SRR1850957 | placenta | Placenta |  |
| *Homo sapiens* | GCF_000001405.40 | SRR1850958 | placenta | Placenta |  |
| *Homo sapiens* | GCF_000001405.40 | SRR1850959 | placenta | Placenta |  |
| *Homo sapiens* | GCF_000001405.40 | SRR1850963 | placenta | Placenta |  |
| *Homo sapiens* | GCF_000001405.40 | SRR1850965 | placenta | Placenta |  |
| *Homo sapiens* | GCF_000001405.40 | SRR1850966 | placenta | Placenta |  |
| *Homo sapiens* | GCF_000001405.40 | SRR1850969 | placenta | Placenta |  |
| *Homo sapiens* | GCF_000001405.40 | SRR1850970 | placenta | Placenta |  |
| *Homo sapiens* | GCF_000001405.40 | SRR1850973 | placenta | Placenta |  |
| *Homo sapiens* | GCF_000001405.40 | SRR1850976 | placenta | Placenta |  |
| *Homo sapiens* | GCF_000001405.40 | SRR1850977 | placenta | Placenta |  |
| *Homo sapiens* | GCF_000001405.40 | SRR1850978 | placenta | Placenta |  |
| *Homo sapiens* | GCF_000001405.40 | SRR1850984 | placenta | Placenta |  |
| *Homo sapiens* | GCF_000001405.40 | SRR1850985 | placenta | Placenta |  |
| *Homo sapiens* | GCF_000001405.40 | SRR1850986 | placenta | Placenta |  |
| *Homo sapiens* | GCF_000001405.40 | SRR1850988 | placenta | Placenta |  |
| *Homo sapiens* | GCF_000001405.40 | SRR1850989 | placenta | Placenta |  |
| *Homo sapiens* | GCF_000001405.40 | SRR1850990 | placenta | Placenta |  |
| *Homo sapiens* | GCF_000001405.40 | SRR1850992 | placenta | Placenta |  |
| *Homo sapiens* | GCF_000001405.40 | SRR1850994 | placenta | Placenta |  |
| *Homo sapiens* | GCF_000001405.40 | SRR1850998 | placenta | Placenta |  |
| *Homo sapiens* | GCF_000001405.40 | SRR1851000 | placenta | Placenta |  |
| *Homo sapiens* | GCF_000001405.40 | SRR1851001 | placenta | Placenta |  |
| *Homo sapiens* | GCF_000001405.40 | SRR1851002 | placenta | Placenta |  |
| *Homo sapiens* | GCF_000001405.40 | SRR1851009 | placenta | Placenta |  |
| *Homo sapiens* | GCF_000001405.40 | SRR649362 | Testis | Testis |  |
| *Homo sapiens* | GCF_000001405.40 | SRR306857 | Testis | Testis |  |
| *Homo sapiens* | GCF_000001405.40 | SRR306858 | Testis | Testis |  |

**
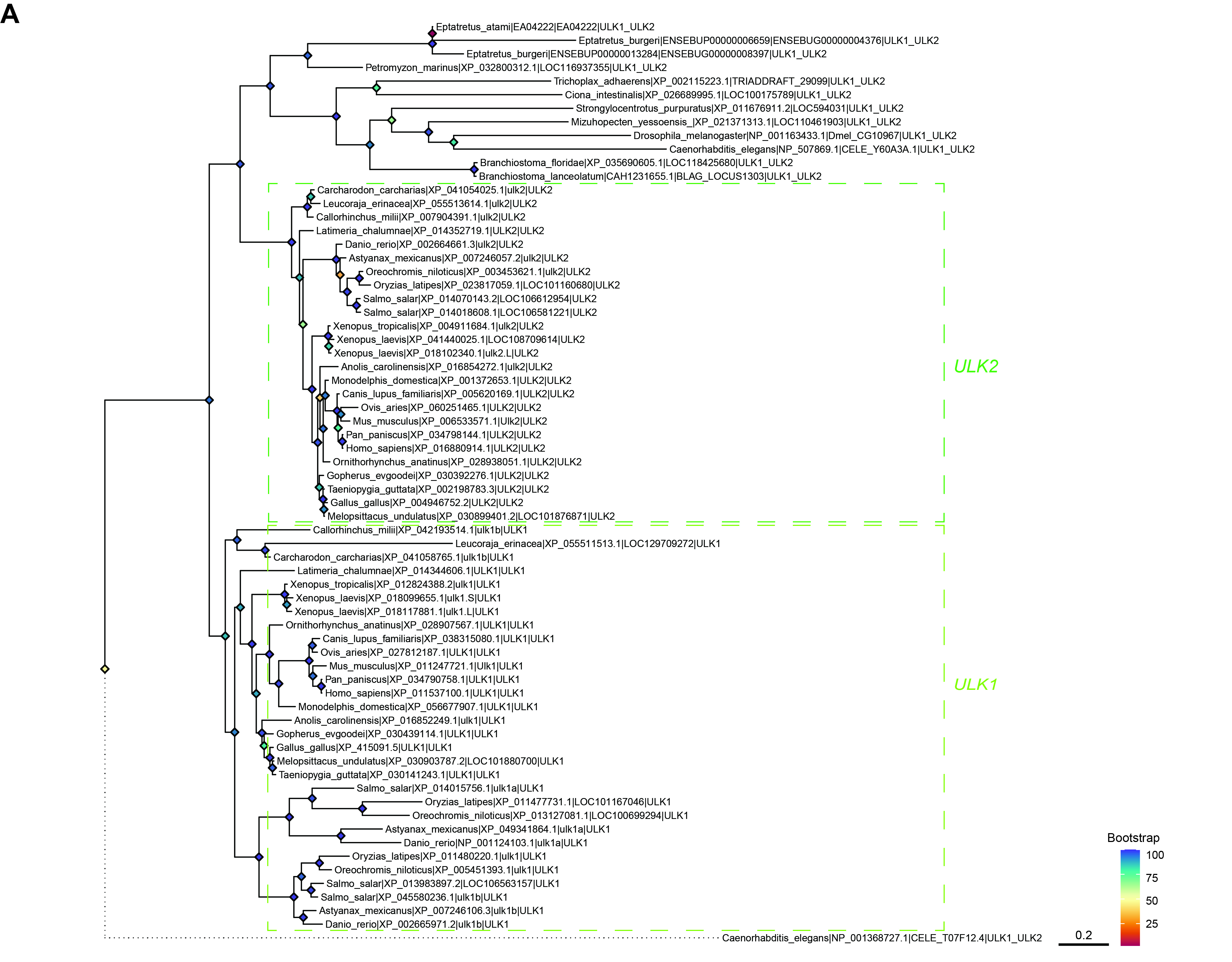
**

**
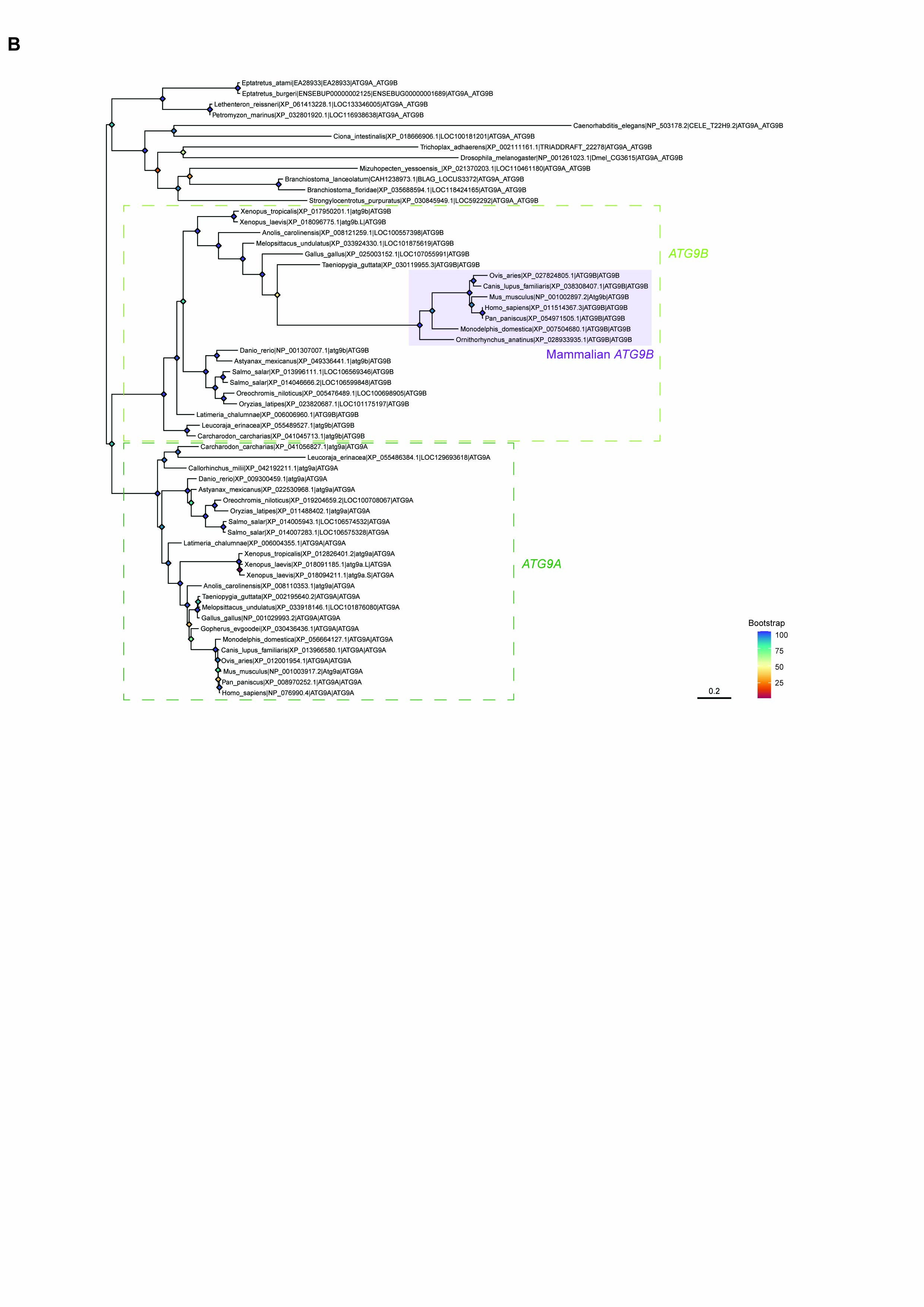
**

**
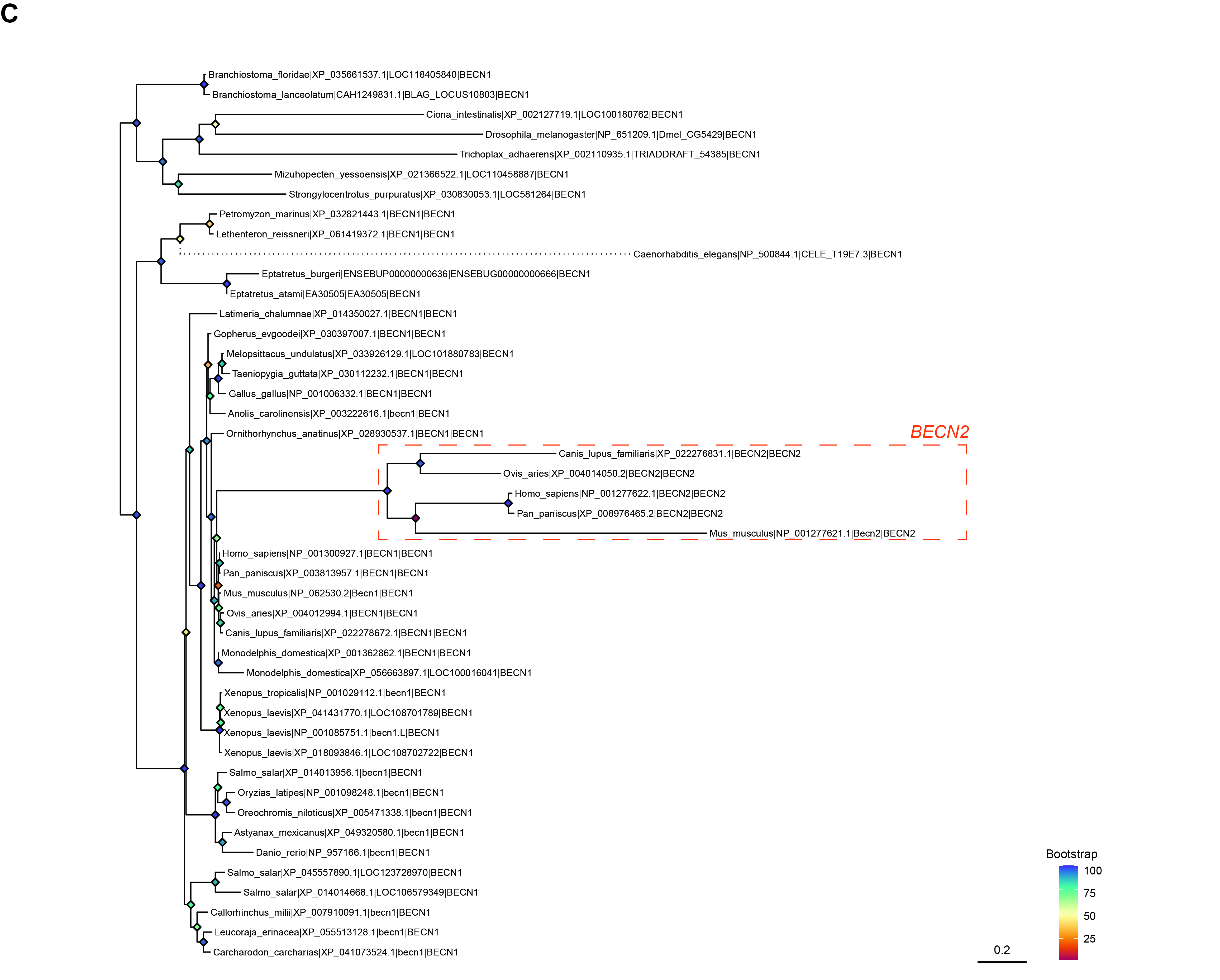
**

**
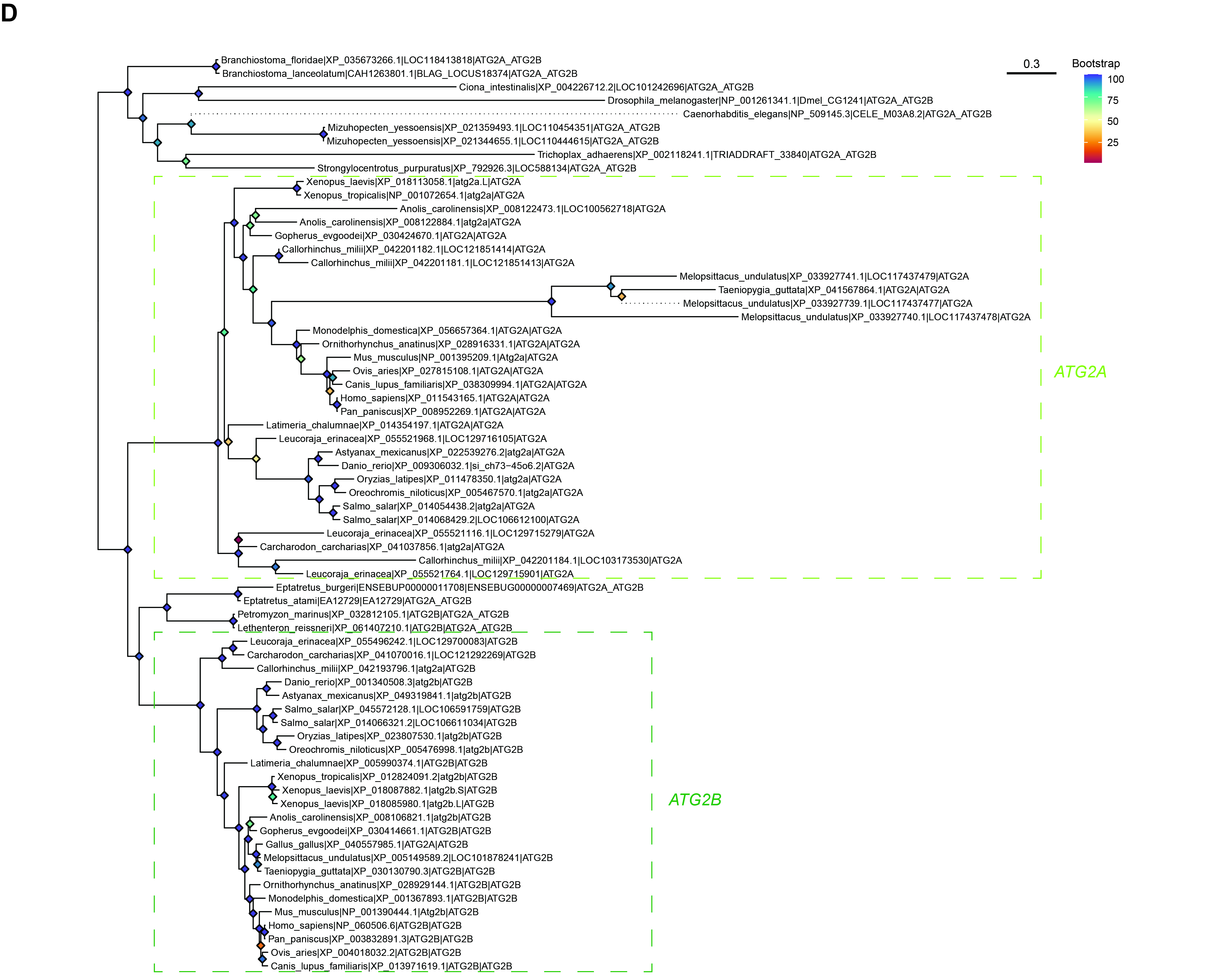
**

**
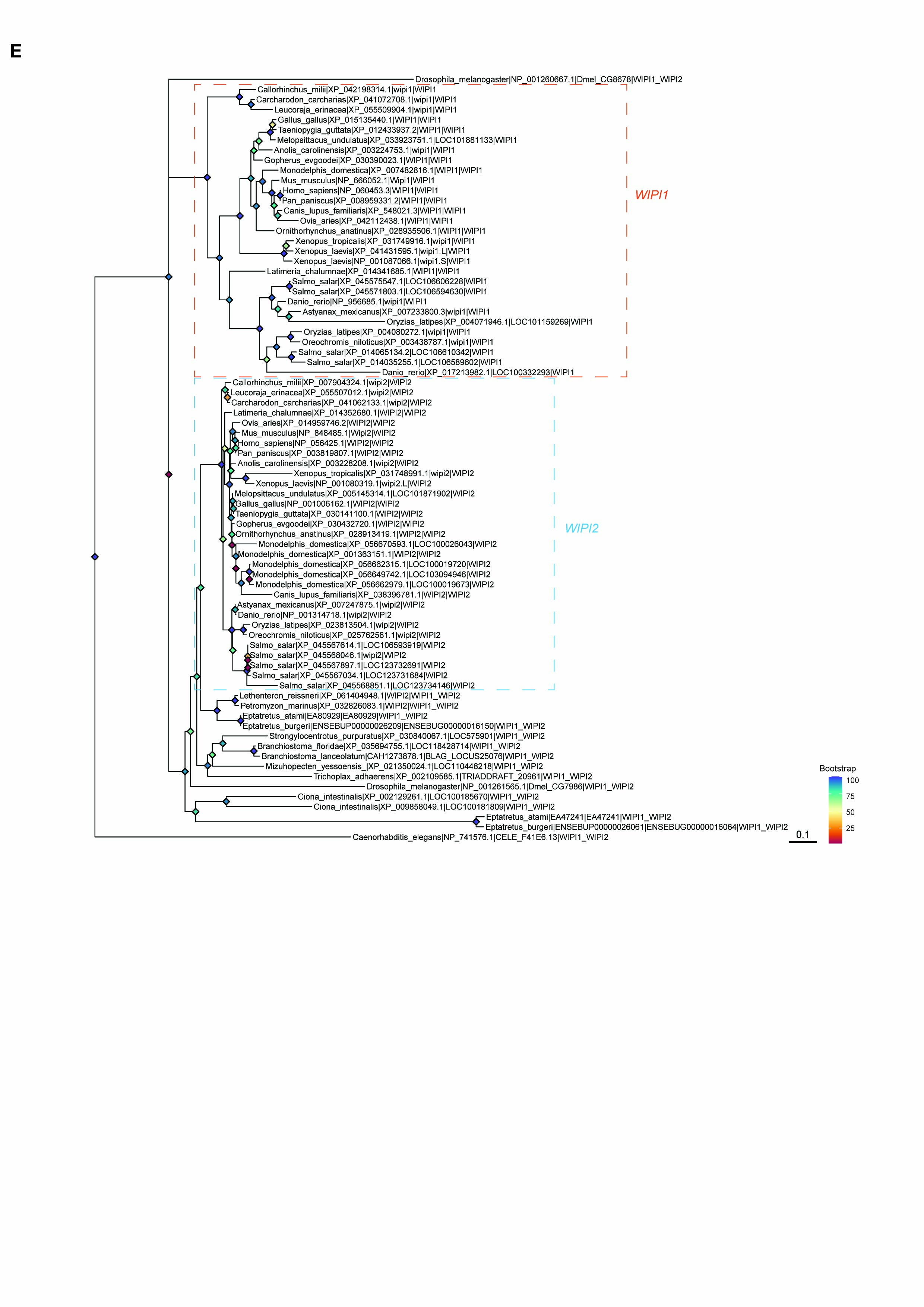
**

**
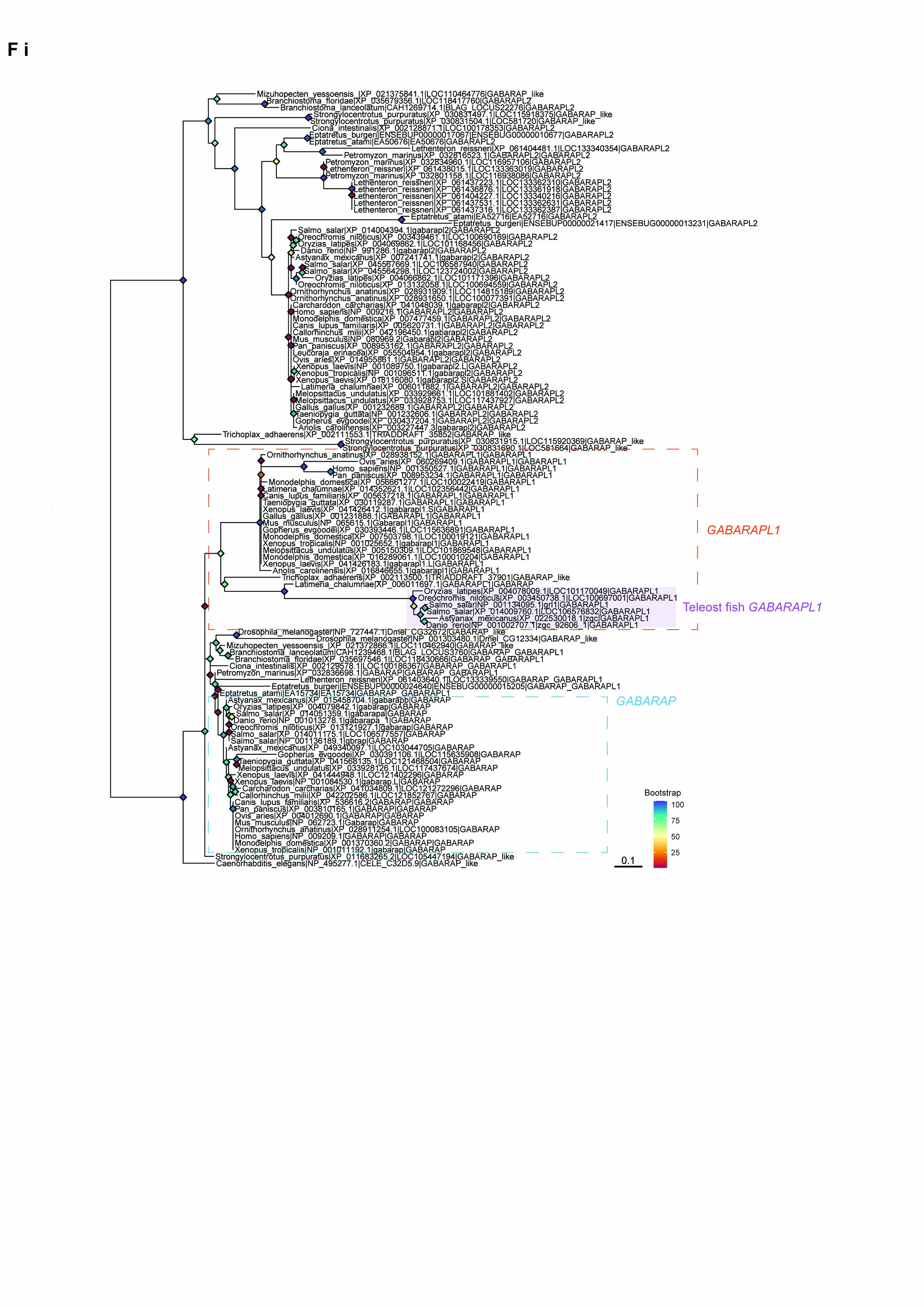
**

**
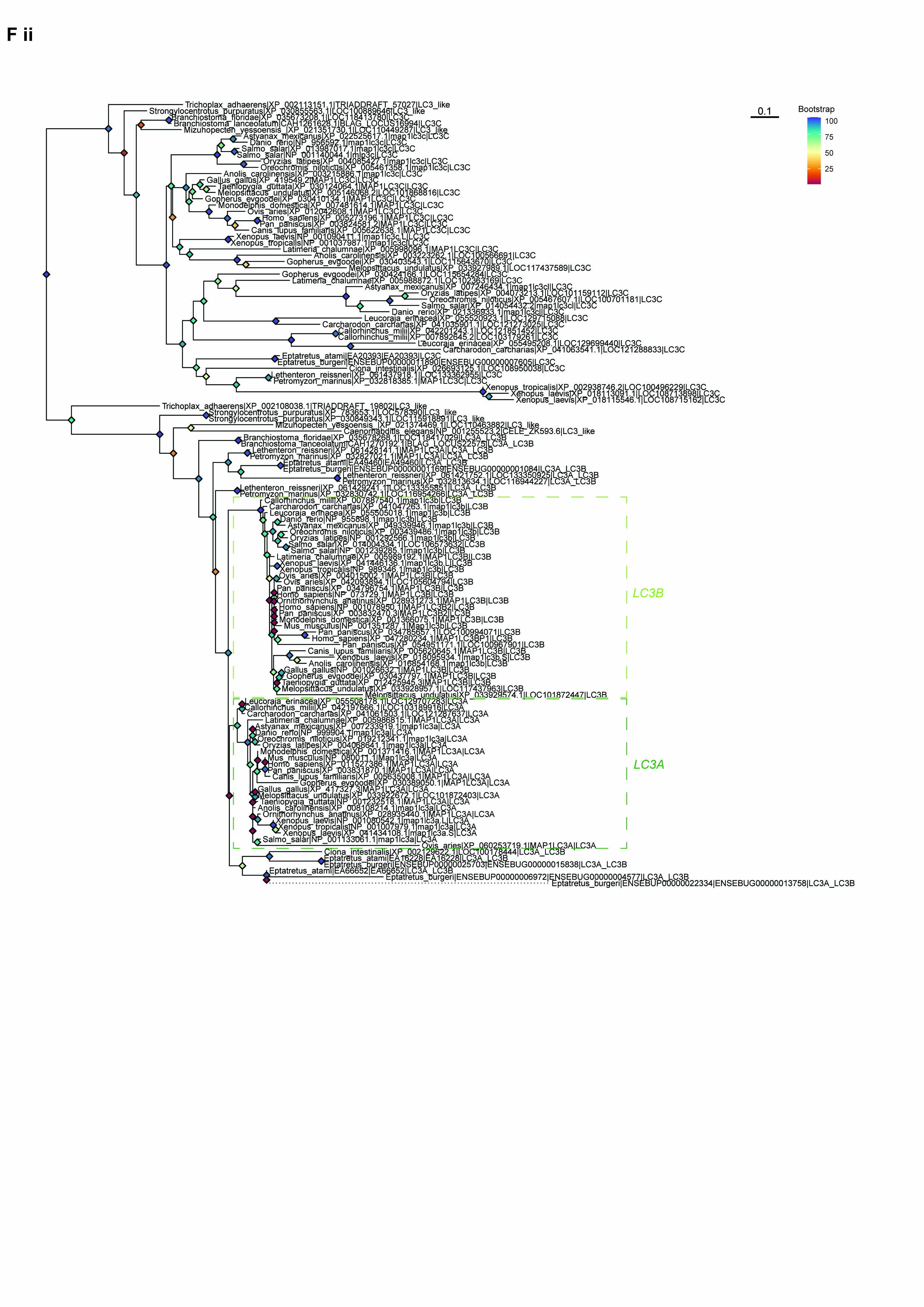
**

**
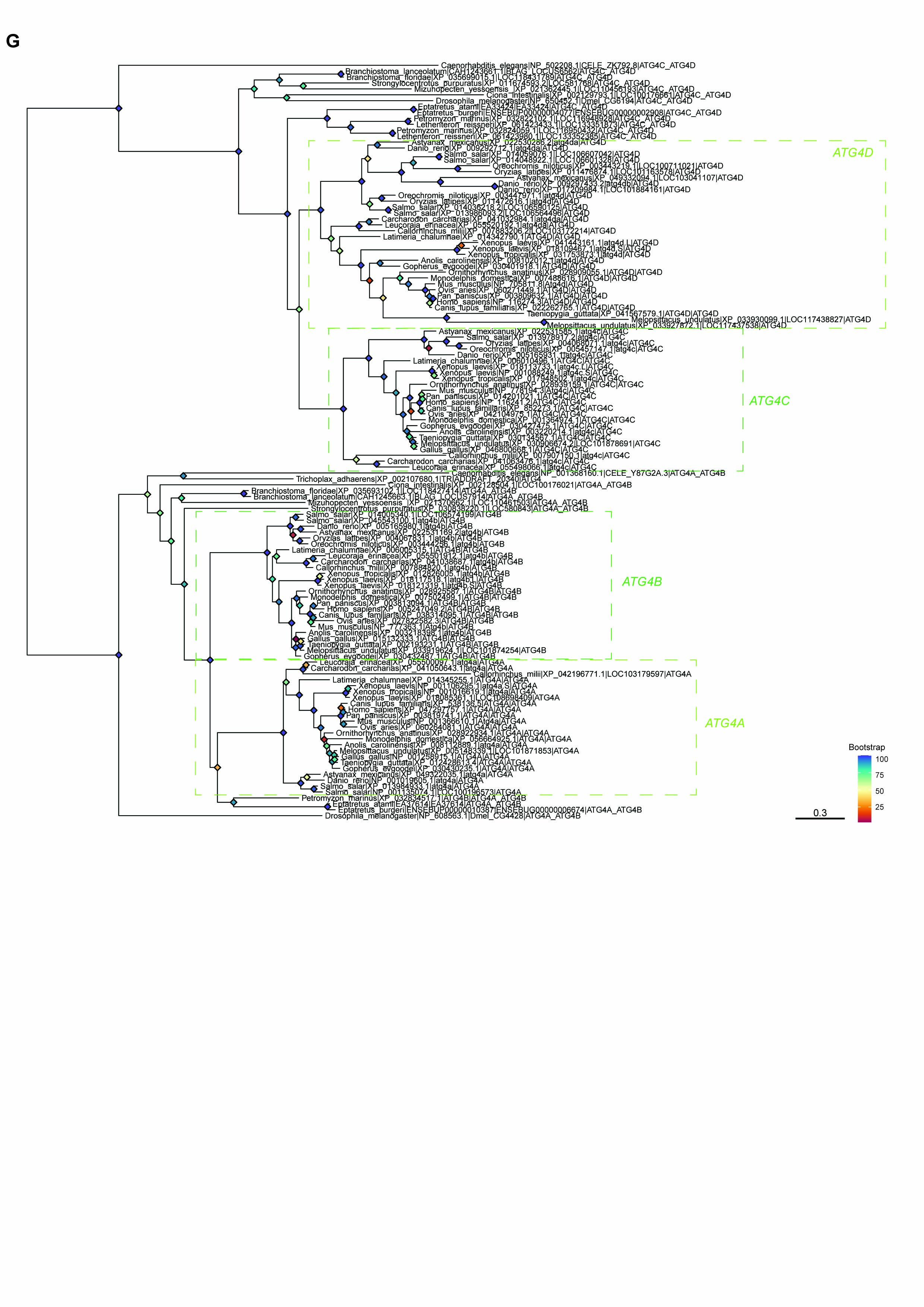
**

**
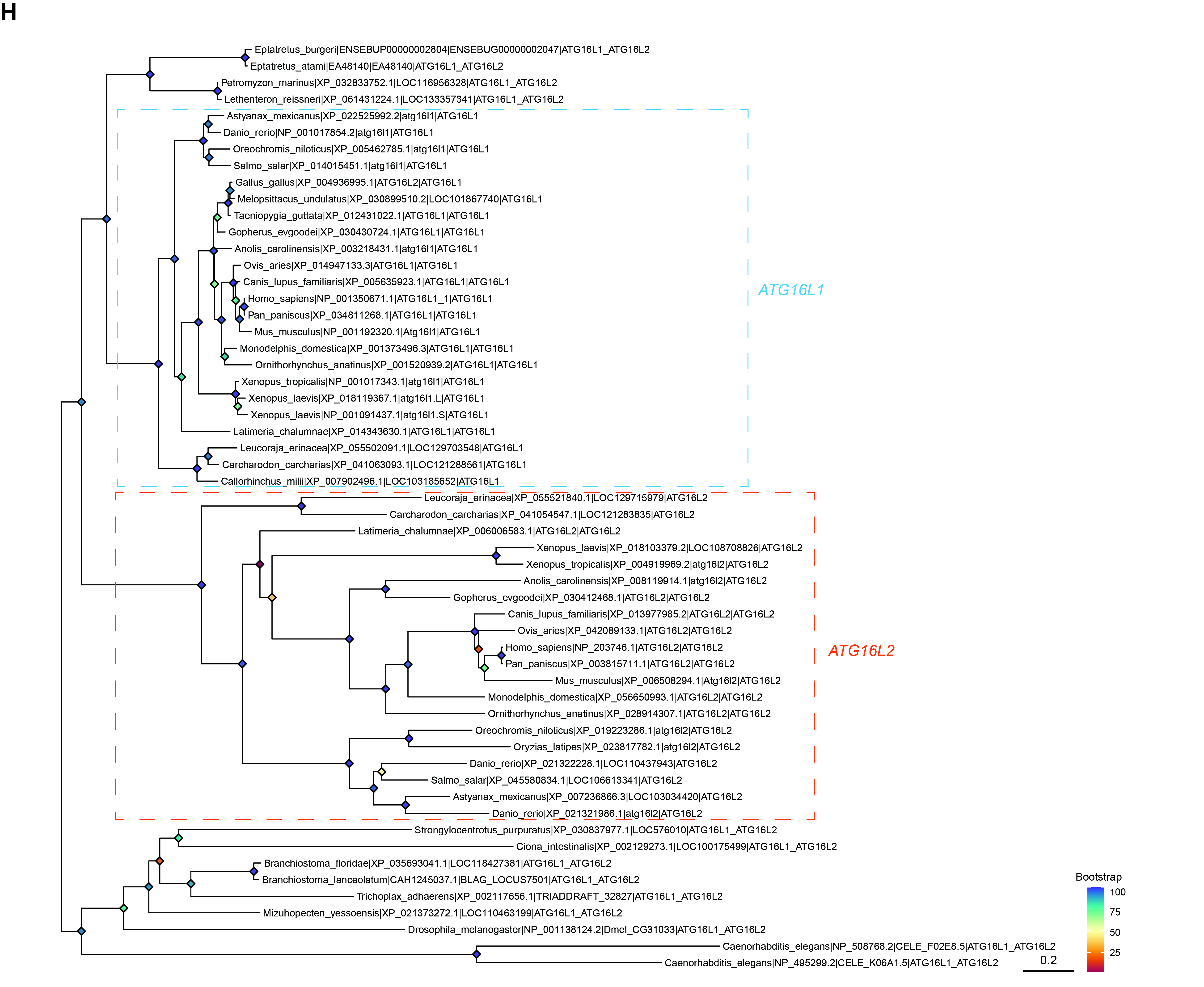
**

**Figure S1**. The phylogenetic trees of (**A**) *ULK-1* (**B**) *ATG9* (**C)** *BECN* (**D**) *ATG2* (**E**) *WIPI* (**F**) *ATG8* (including **i**, *GABARAP*s and **ii**, *LC3*s) (**G**) *ATG4*, and (**H**) *ATG16* homologs before and after gene duplications, manually rooted with five outgroup species, namely *Drosophila melanogaster*, *Caenorhabditis elegans*, *Strongylocentrotus purpuratus* (purple sea urchin), *Mizuhopecten yessoensis* (scallop), and *Trichoplax adhaerens*. Bootstrap values of the nodes are indicated by their color. Dotted lines indicate long branches that were manually shrunk out of proportion for display purposes. Mammalian *ATG9B* sequences and teleost fish *GABARAPL1* sequences on long branches are highlighted in purple. *BECN2* sequences (orange rectangle) have only been found in eutherians.

**
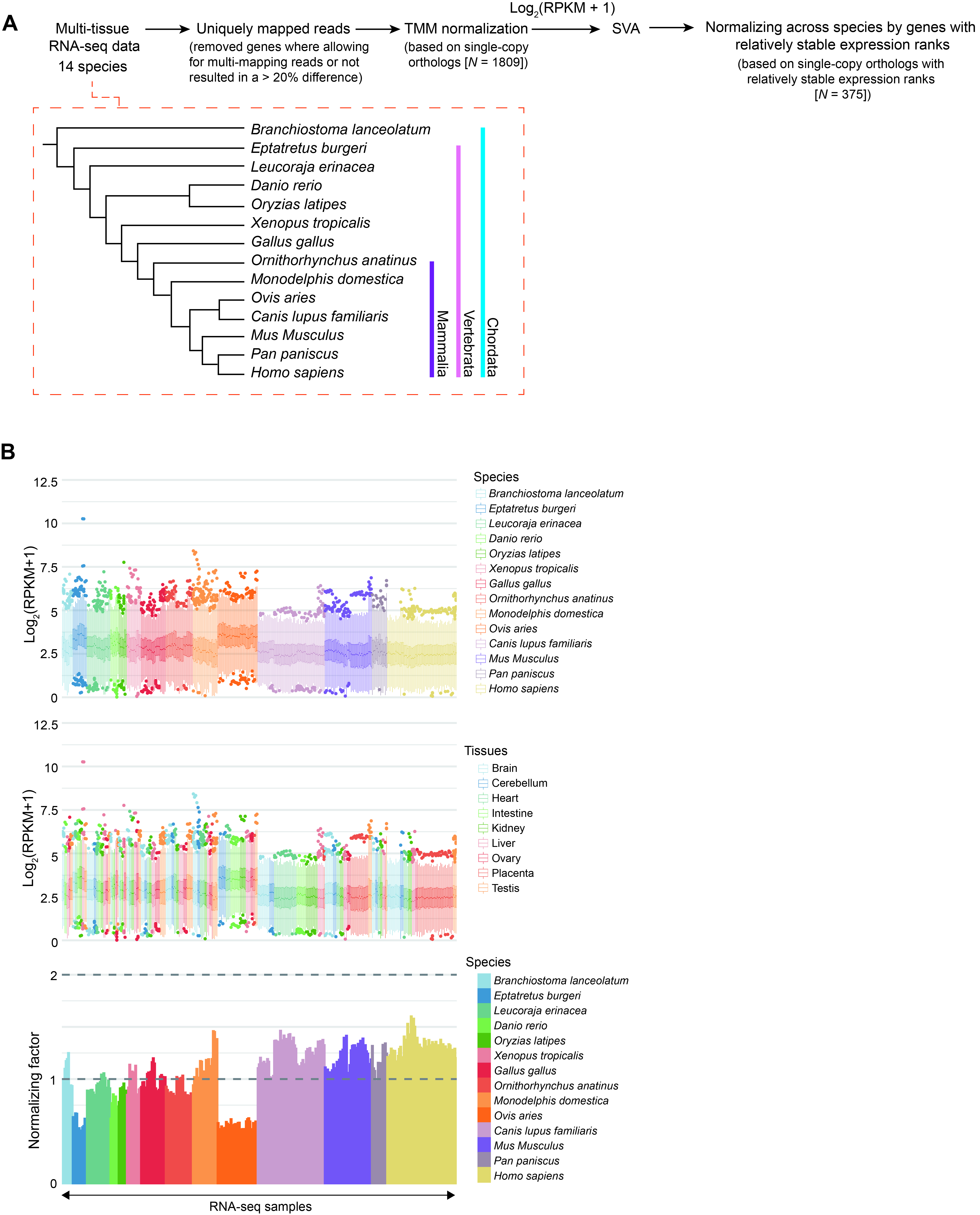
**

**
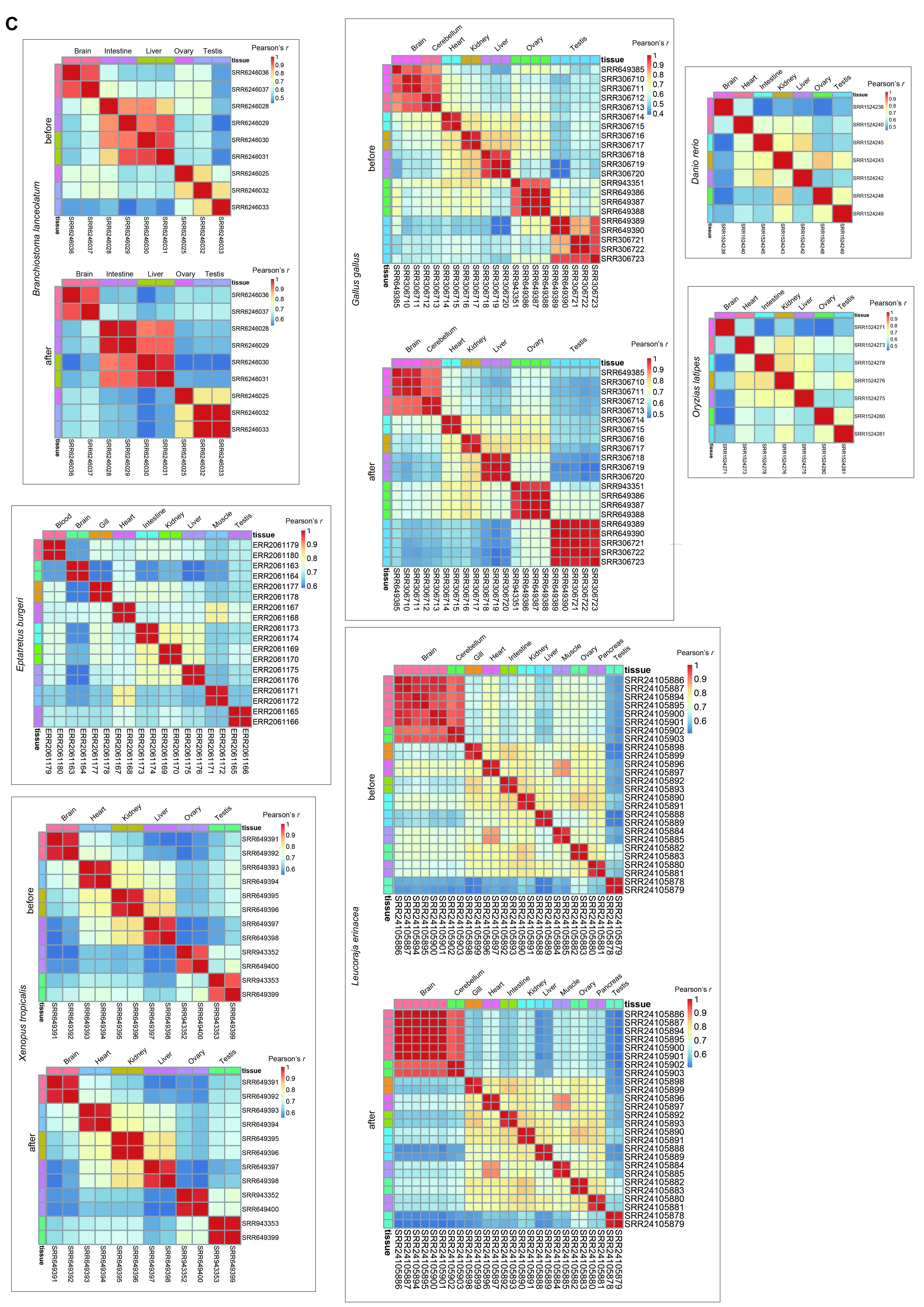
**

**
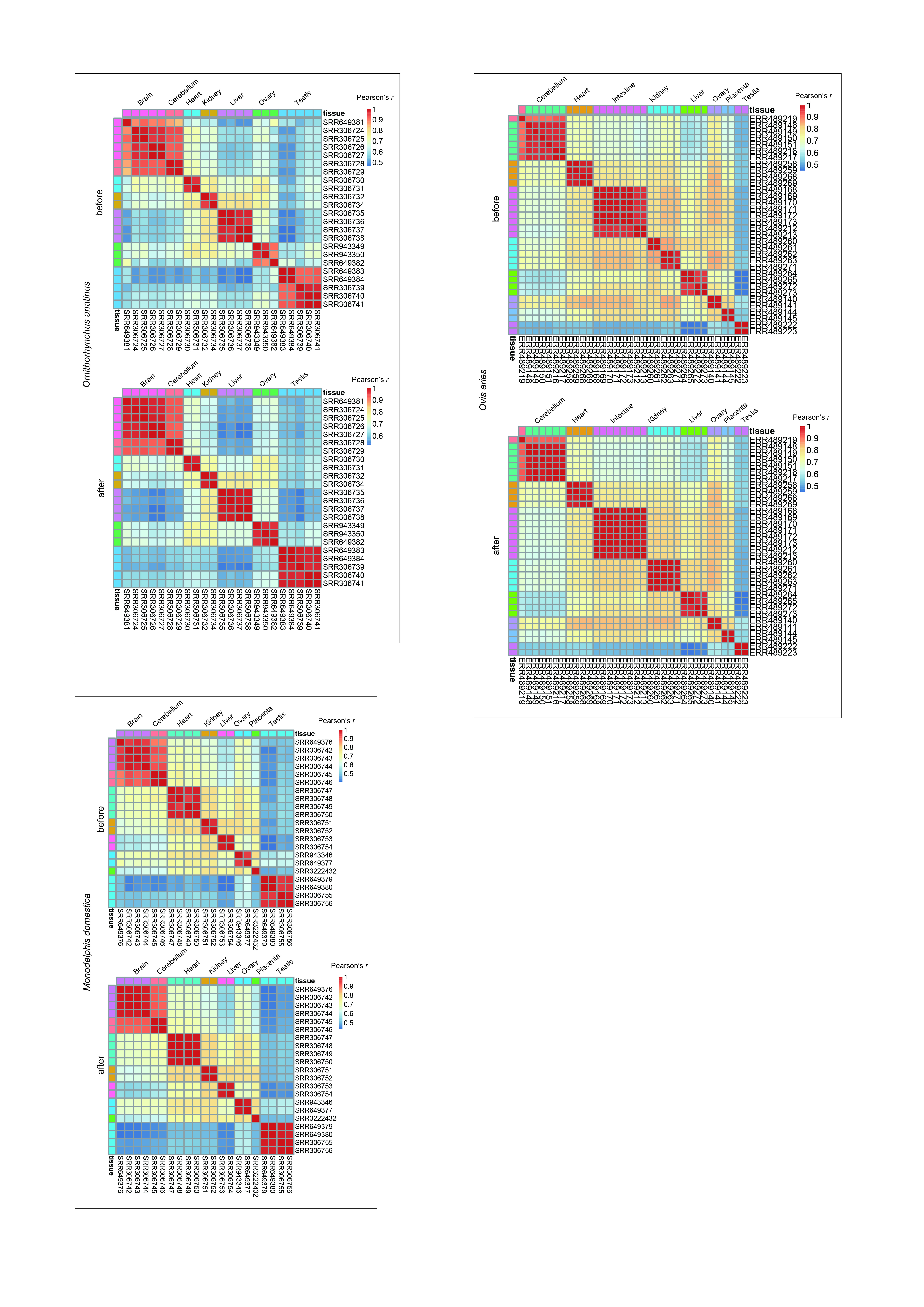
**

**
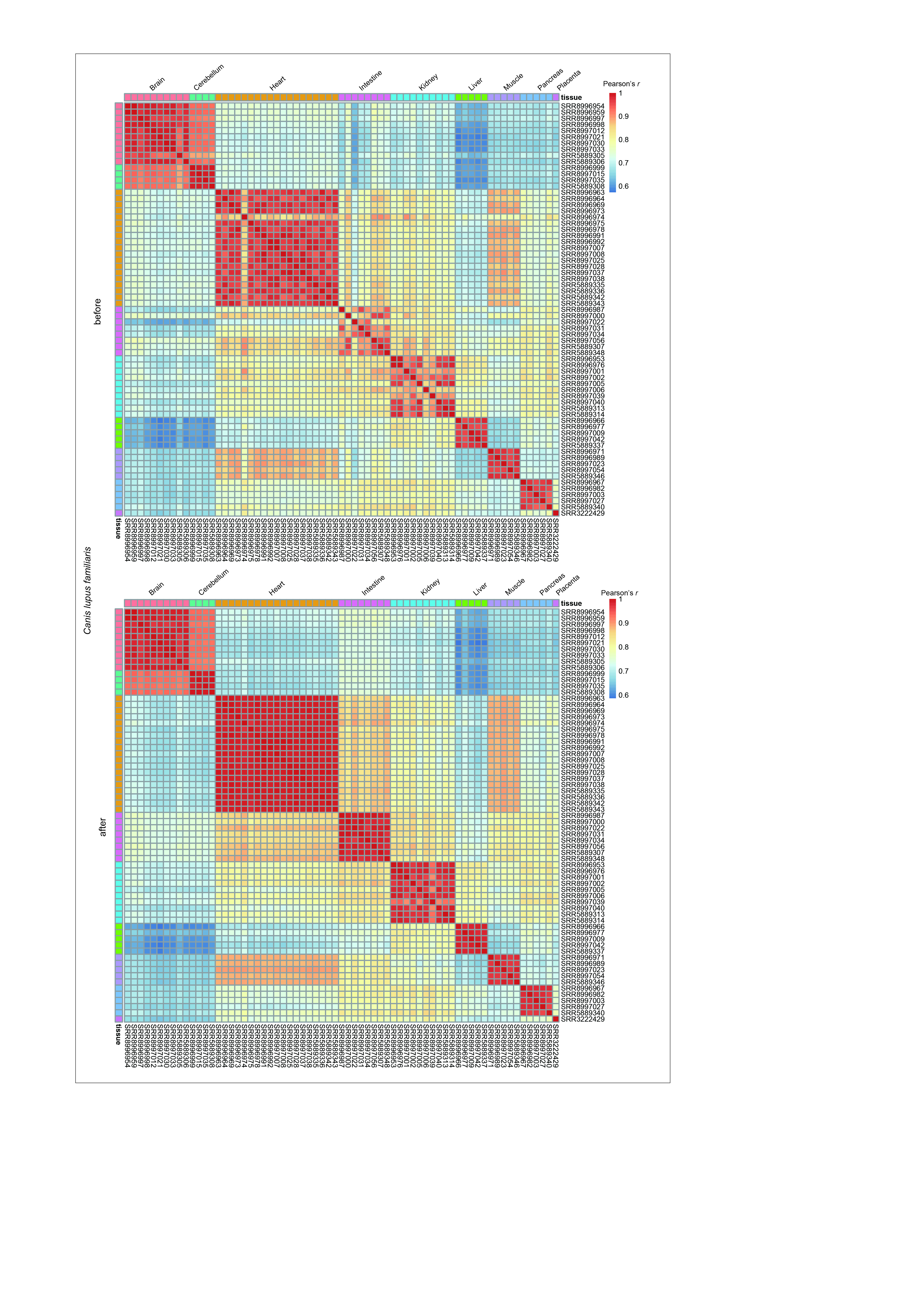
**

**
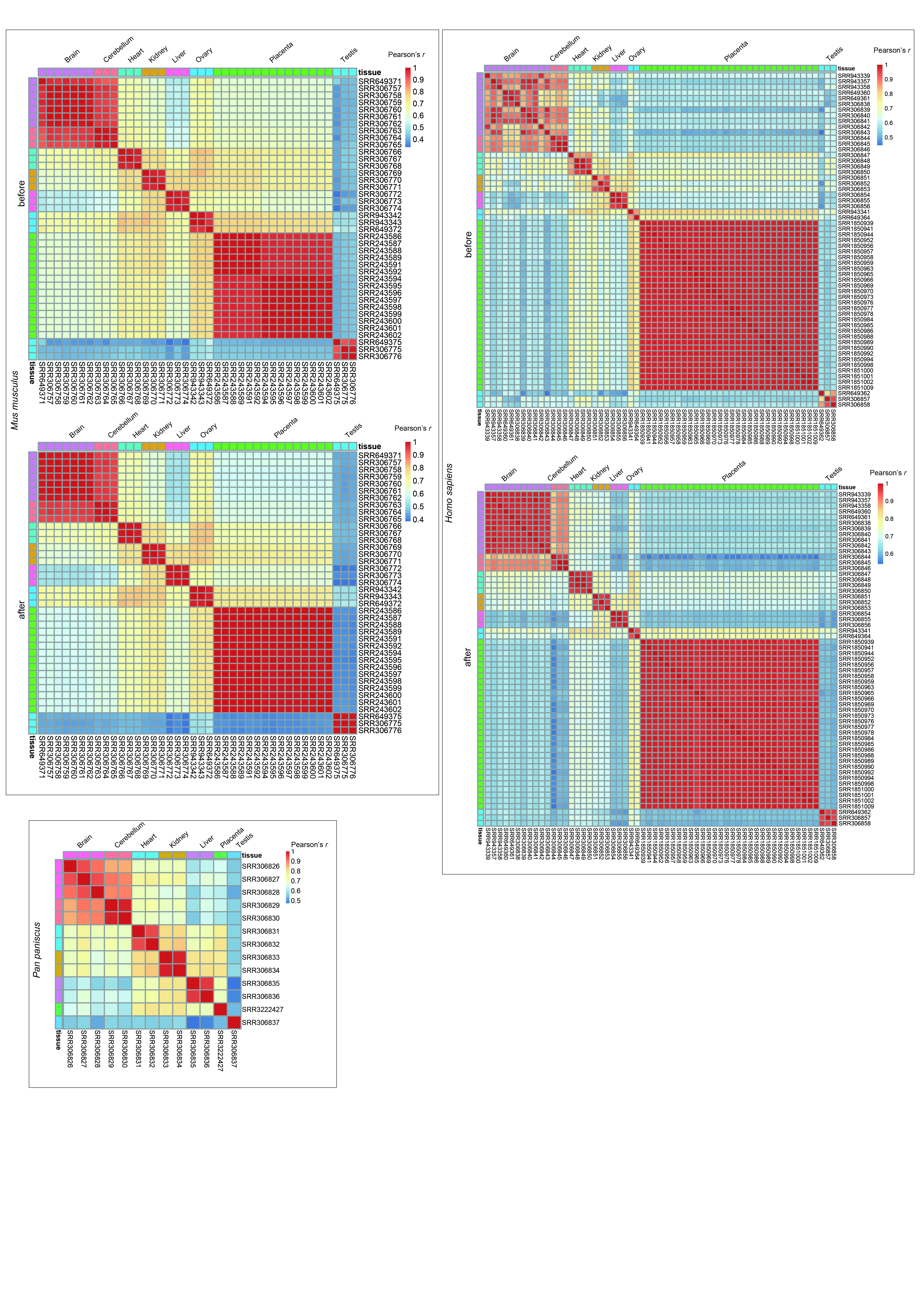
**

**Figure S2**. The quality control and normalization procedures for the RNA-seq data were applied to ensure that gene expression levels would be comparable among samples and species. (**A**) The overall quality control and normalization pipeline. Only the uniquely mapped reads were used in the analysis after removing genes in which counting all reads or counting only the uniquely mapped reads resulted in a >20% difference. In addition to the TMM normalization and the standard log_2_(RPKM+1) transformation, SVA was applied to remove hidden technical variables, and gene expression levels were further normalized based on the median of 375 single-copy orthologs with relatively stable expression ranks across samples and species. (**B**) The expression levels of 375 single-copy orthologs with relatively stable expression ranks across samples and species, colored by species (top) and tissue type (middle), and the normalizing factors calculated based on the median expression levels of these genes (bottom; all other samples were normalized to the leftmost sample). (**C**) SVA increased Pearson’s *r* coefficient between samples of the same tissue types (calculated using all available genes). In each heatmap, each row and column represents one sample, and the samples are grouped by tissue type in a predefined order. For species in which SVA was not applied (see Materials and Methods for details), Pearson’s *r* without SVA is shown.

**
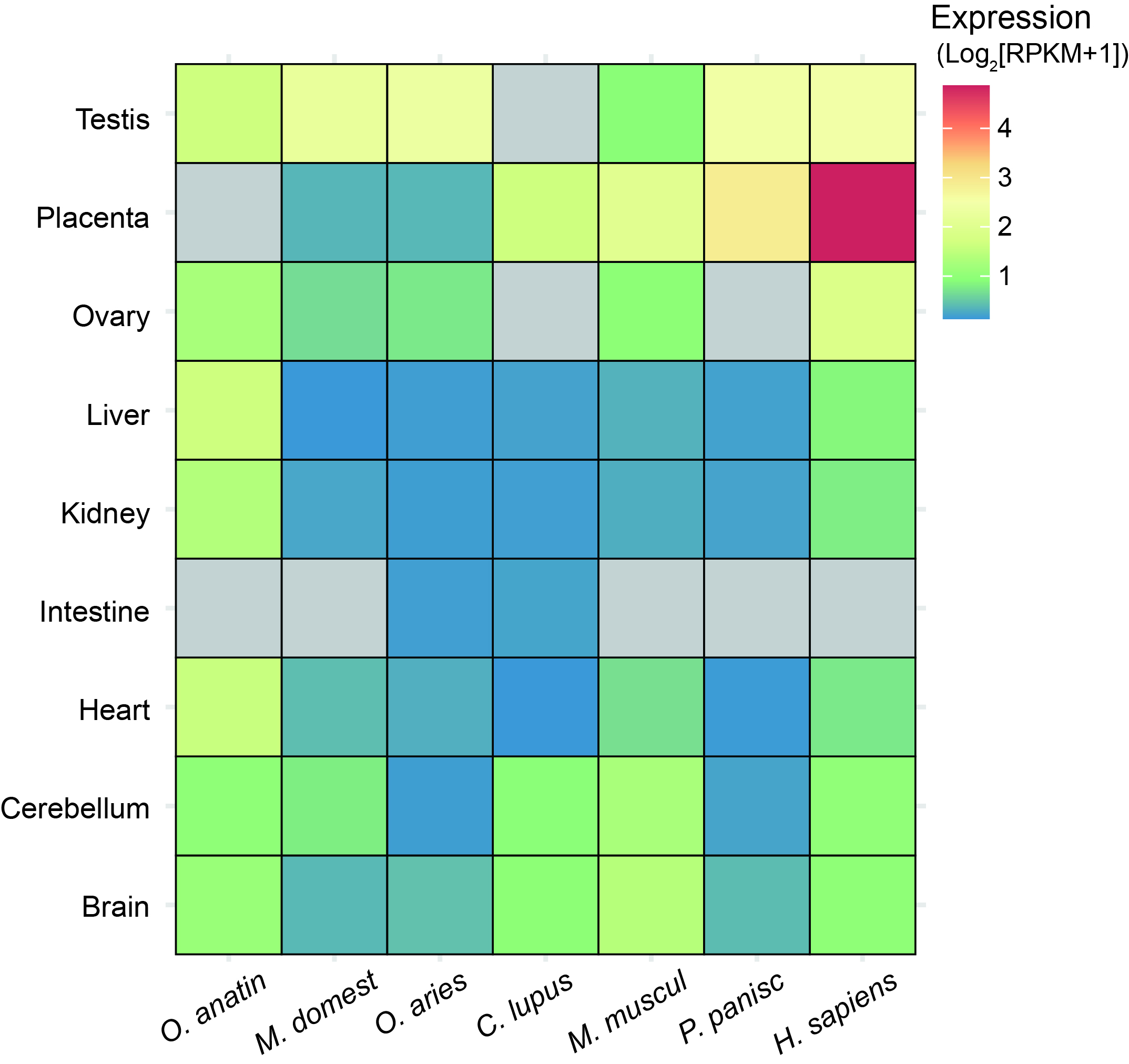
**

**Figure S3**. *ATG9B* has placenta- or testis-enriched expression in mammals. Color indicates the expression levels; gray specifically means no data were available. *O. anatin*, *Ornithorhynchus anatinus*; *M. domest*, *Monodelphis domestica*; *O. aries*, *Ovis aries*; *C. lupus*, *Canis lupus familiaris*; *M. muscul*, *Mus musculus*; *P. panisc*, *Pan paniscus*; *H. sapiens*, *Homo sapiens*.

**
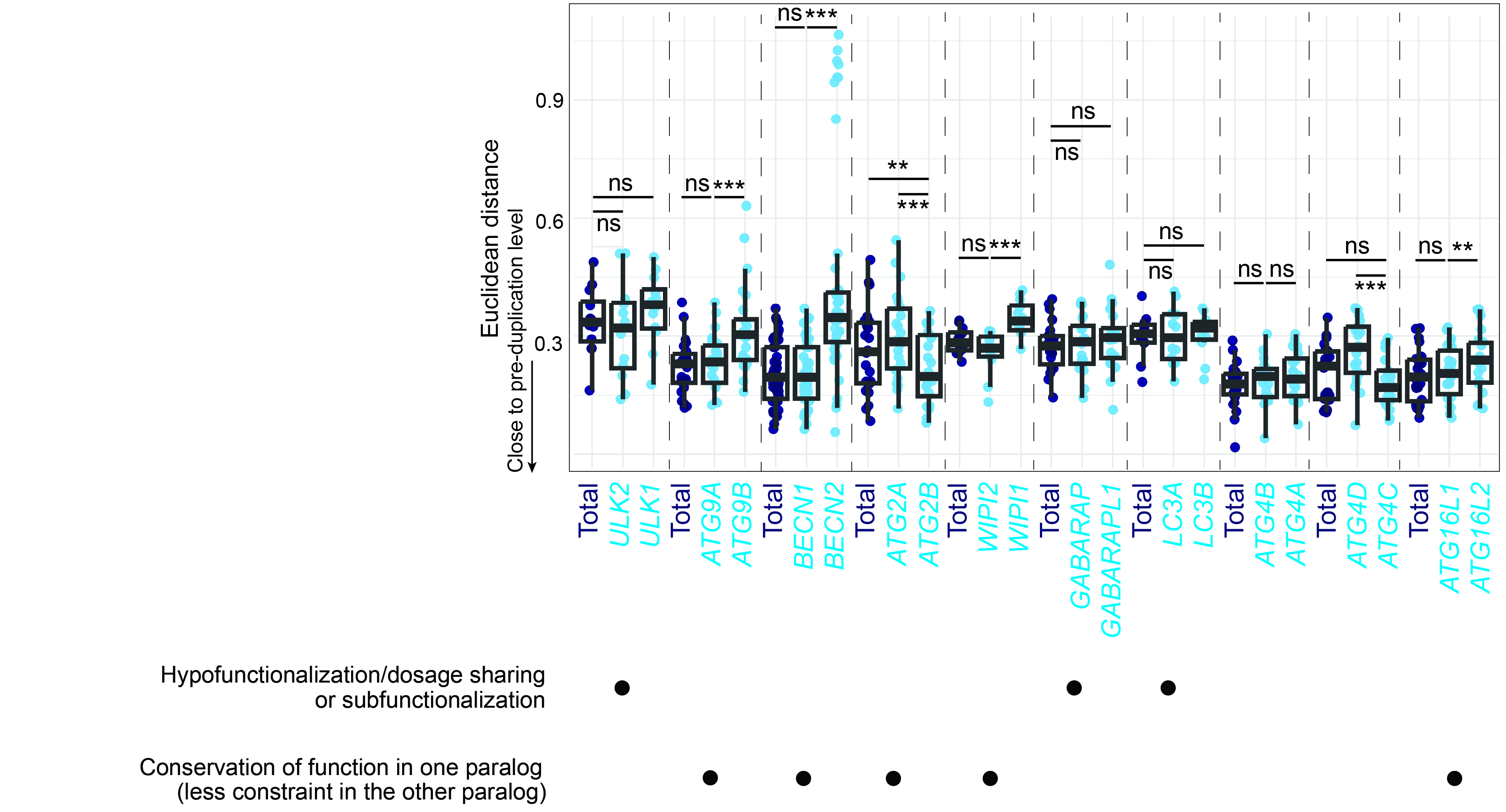
**

**Figure S4**. Evolutionary fate classification of the *ATG* paralogs based on the relative gene expression levels (gene expression in each tissue normalized to the total expression across all tissues), with associated evolutionary fate categories shown below. A generalized linear mixed model was used to compare the differences in Euclidean distances. *, *p* < 0.05. **, *p* < 0.01. ***, *p* < 0.001. ns, non-significant (*p* > 0.05).
